# Promoting interactive skills and mind-mindedness among early childcare professionals: study protocol for a randomized wait-list controlled trial comparing the Circle of Security approach with care as usual in center-based childcare (the SECURE project)

**DOI:** 10.1186/s40359-022-00835-3

**Published:** 2022-06-18

**Authors:** Johanne Smith-Nielsen, Katrine Isabella Wendelboe, Julie Elisabeth Warberg Mohr, Mette Skovgaard Væver, Maiken Pontoppidan, Katrien Helmerhorst, Ida Egmose

**Affiliations:** 1 Department of Psychology, University of Copenhagen, Øster Farimagsgade 2A, 1353 Copenhagen, Denmark; 2 Department of Health, VIVE—The Danish Center for Social Science Research, Herluf Trolles Gade 11, 1353 Copenhagen, Denmark; 3 Department of Pedagogy and Educational Sciences, Child and Family Welfare, University of Groningen, Grote Rozenstraat 38, 9712 TJ Groningen, The Netherlands

**Keywords:** Center-based childcare, Attachment-based intervention, Circle of Security, COSP-Classroom, Caregiver–child interaction quality, Early childhood education, Process quality, Interactive skills, Mind-mindedness, Mentalization

## Abstract

**Background:**

In countries where the majority of young children are enrolled in professional childcare, the childcare setting constitutes an important part of children’s caregiving environment. Research consistently shows that particularly the quality of the daily interactions and relationship between young children and their professional caregivers have long-term effects on a range of developmental child outcomes. Therefore, professional caregivers’ capacity for establishing high quality interactions with the children in their care is an important target of intervention.

**Methods:**

A prospective, parallel, cluster-randomized wait-list controlled trial is used to test the efficacy of the attachment- and mentalization theory informed Circle of Security (COS) approach adapted to the childcare setting (COS-Classroom) on caregiver interactive skills and mind-mindedness. Participants are professional caregivers of children aged 0–2.9 years working in center-based childcare in Denmark. Approximately 31 childcare centers, corresponding to an estimated 113 caregivers, are expected to participate. The primary outcome is caregiver Sensitive responsiveness measured with the Caregiver Interactive Profile Scales (CIP-scales). Secondary outcomes include caregiver Mind-mindedness, the five remaining CIP-scales (Respect for autonomy, Structure and limit setting, Verbal communication, Developmental stimulation, and Fostering positive peer interactions), and caregivers’ resources to cope with work-related stress. Data on structural factors (e.g., staff stability, caregiver-child ratio, and level of pre-service education), caregiver attachment style, acceptability and feasibility of the COS-C together with qualitative data on how the participants experience the COS-C is additionally collected to investigate moderating and confounding effects.

**Discussion:**

Examining the effectiveness of the COS-C in center-based childcare contributes to the knowledge of evidence-based intervention programs and can potentially improve the caregiver quality early childcare.

*Trial registration*: ClinicalTrials.gov: NCT04654533. Prospectively registered December 4, 2020, https://clinicaltrials.gov/ct2/show/NCT04654533.

## Background

It is well-established that the quality of the early caregiving environment, and specifically the quality of the interaction and the relationship between young children and their primary caregiver(s) are positively associated with a wide array of children’s social, emotional and cognitive outcomes (e.g. [1–3].). Caregivers thus play a pivotal role in determining quality of care for children’s development. In Denmark, where the current study is conducted, the majority of children younger than three years are enrolled in professional childcare more than 30 h per week [4]. Therefore, the childcare setting constitutes an important part of children’s early caregiving environment, and following, childcare quality becomes important in terms of promoting healthy child development.

When defining ‘quality of care’ in childcare, a distinction between *structural* and *process* quality is typically made [5]. Structural quality refers to characteristics such as caregiver-child ratio, group size, caregiver educational level, and caregiver stability. Process quality refers to the quality of experiences and interactions (with caregivers, other children, materials, and parents) children have within the childcare settings [5]. Whereas structural quality is considered a prerequisite of process quality and as such of indirect effect on children’s developmental outcomes, process quality is thought to directly affect young children’s well-being and learning as well as long-term healthy social, emotional, and cognitive development [6–10]. The importance of process quality for children’s development is stressed by the results from a recent meta-analysis of 17 longitudinal studies including 16,461 children from nine European countries. This review showed that high-level process quality in early childcare has a significant positive and lasting association on children’s language and cognitive development irrespectively of family background and SES [11].

In early childcare (i.e. children younger than 3 years old) the relationship and the daily interactions between children and their caregivers are considered the most important aspect of process quality [12, 13]. In both high and low SES populations studies have found that young children’s development and well-being is directly linked with caregiver-child interaction quality [14–17]. While all children benefit from high quality caregiver-child interactions [5–8], (positive and negative) effects of the caregiver-child relationship are strongest for children at higher risk for adversity [9, 18–20].

In Denmark, a number of interventions have been implemented in center-based childcare to promote healthy development in children coming from at-risk backgrounds [21]. However, the majority of the interventions focus on improving specific child skills such as language acquisition or motor development [22, 23]. As studies show that the quality of the caregiver-child relationship can act as a protecting factor in vulnerable children’s lives [9, 18–20] this is an important target of intervention in terms of preventing adverse child outcomes.

A recent meta-analysis examining the effects of interventions focusing on professional caregivers’ relational capacity and interactive skills on the caregiver-child interaction quality found a moderate positive effect on overall caregiver-child interactions (*k* = 19, Hedges’ g = 0.35) [24]. While the authors conclude that intervention programs in early childcare may lead to higher childcare quality, they also stress that there is a need for more well-designed randomized controlled trials of various interventions to shed light on which intervention programs are most effective in terms of promoting interaction quality and supporting healthy development in children.

Therefore, the overall objectives of this study are to examine the effect, acceptability, and feasibility of a professional development intervention based on the Circle of Security approach [25] offered to Danish caregivers working in childcare centers with children aged 0–3 years old.

### Caregiver interactive skills and sensitivity

Caregiver sensitivity refers to the extent to which a caregiver recognizes children’s individual emotional and physical needs and responds appropriately and promptly to their cues and signals [26] and is the key aspect of caregiving in attachment theory [27, 28]. Parental sensitivity is known to be predictive of a range of positive child outcomes, such as attachment security, language, cognitive, and socio-emotional functioning [29]. Accordingly, sensitivity is considered a key element of professional childcare quality [30]. Indeed, professional caregivers who are responsive to the children’s need for comfort, closeness, and regulation during distress and at the same time stimulate the children’s exploration and provide opportunities to learn, are generally considered as providing high-process-quality childcare [13, 31, 32]. Therefore, caregiver sensitivity is an important target for interventions in childcare and it is the primary outcome in the current study.

It may be challenging for a professional caregiver to stay sensitive to children who have been exposed to low-quality parental care [33]. Children from families at higher risk of adversity are more likely to enter childcare with so-called insecure attachment strategies developed in the interactions with their primary caregiver(s). Child-caregiver attachment quality reflects the emotional bond between a child and a specific caregiver, and is defined by the child’s tendency to seek comfort, help and protection in situations perceived by the child as uncertain, threatening, or in other ways distressing [28]. A child’s attachment relationship to its primary caregiver forms this child’s expectations and behavioral strategies when interacting with new caregivers [28], and a child who has developed insecure attachment strategies to the primary caregiver may show externalizing behavior or social withdrawal when in need of comfort and support [34]. For the caregiver, this may in turn may make it more difficult to interpret and meet the child’s needs as compared with (secure) children who seek comfort and help when needed [28, 35]. Accordingly, it has been documented that the quality of the relationship between children and their professional caregivers often corresponds to the quality of the parent–child attachment relationship [33], thereby putting children who are insecurely attached to its primary caregivers at ‘double risk’. Indeed, children exposed to low quality parental and low quality childcare are consistently found to have the worst developmental outcomes [36–38].

While research thus suggests that a child’s interactive strategies may be reproduced in interactions with new caregivers, meta-analytic evidence also shows that the interactive behavior, and specifically the caregiver’s ability to stay sensitive to childrens emotional needs, predicts the quality of the relationship between children and their professional caregivers independently of the children’s relationhip quality to their primary caregivers [33]. In other words, if the professional caregiver is able to stay sensitive to a child who is insecurely attached to its primary caregiver, it is possible for the child to develop a secure relationship to the professional caregiver regardless of the quality of the child’s attachment to the primary caregiver. An important focus of the intervention tested in the current study is to support caregivers in staing sensitive and responsive to children who may not display their emotional needs clearly, i.e. to understand “the need behind the behavior”.

### Caregiver mindmindedness

A caregiver’s capacity to treat the child as a psychological individual, i.e. the capacity for mentalizing [39], is thought to be a prerequsite for a caregiver’s ability to be sensitive to children’s needs [40]. It is generally accepted that the ability to mentalize may be linked with both the current state (e.g., stress) and/or more stable factors, such as the caregiver’s own attachment experiences [40]. Particularly in situations where the child and/or the caregiver is distressed, the ability to mentalize is considered essential for continuously providing sensitive caregiving [39, 41]. Keeping a mentalizing stance towards the child enables the caregiver to interpret the child’s behavior in terms of emotional needs. This ability is particularly important when interacting with children who are distressed, socially withdrawn, or in the early phases of language development and interpretation of behavior is the means to understanding the child’s needs [39, 41].

The concept “Mind-Mindedness” (MM) is one way that mentalizing has been operationalized [42]. MM is defined as the caregiver’s tendency to treat the child as a psychological individual with a mind of its own and to “tune-in” to the child’s perspective, while being aware that internal states motivate the child’s actions [43]. While research shows that MM is associated with observed (sensitive) interactive behavior in parents [44], the quality of the parent–child attachment relationship, as well as child developmental outcomes [45, 46], studies on the links between MM and interactive skills in professional caregivers are scarce. However, the existing studies indicate a positive association between MM and sensitive behavior [47–49] and between MM and caregiver emotional involvement [50].

In sum, this research suggests that together with sensitivity, MM/the ability to mentalize is an important focus of intervention in the childcare setting.

### The Circle of Security classroom approach

In the current project, we test the effect of the COS-Classroom model (COS-C). COS-C is adapted from the parenting program, the Circle of Security-Parenting (COSP) [25] and modified to fit the professional childcare context [51]. COS-C is an attachment theory informed professional-development program. It is a manualized eight-session group intervention for caregivers of children aged 0–5 years that combines psycho-education with a mentalization-based approach [51]. The COS framework aims to facilitate caregiver sensitive responsiveness, emotional availability and caregiver emotion regulation, and a core assumption of the model is that enhanced reflective capacity (mentalizing) is the mechanism through which caregiver sensitivity improves which, ultimately, is the key to support secure attachment relationships between caregivers and children [25]. Increased reflection is assumed to be closely related to caregivers’ understanding of how their own relational history and emotion regulation strategies may affect their relationship with the children in their care.

**Fig. 1:**
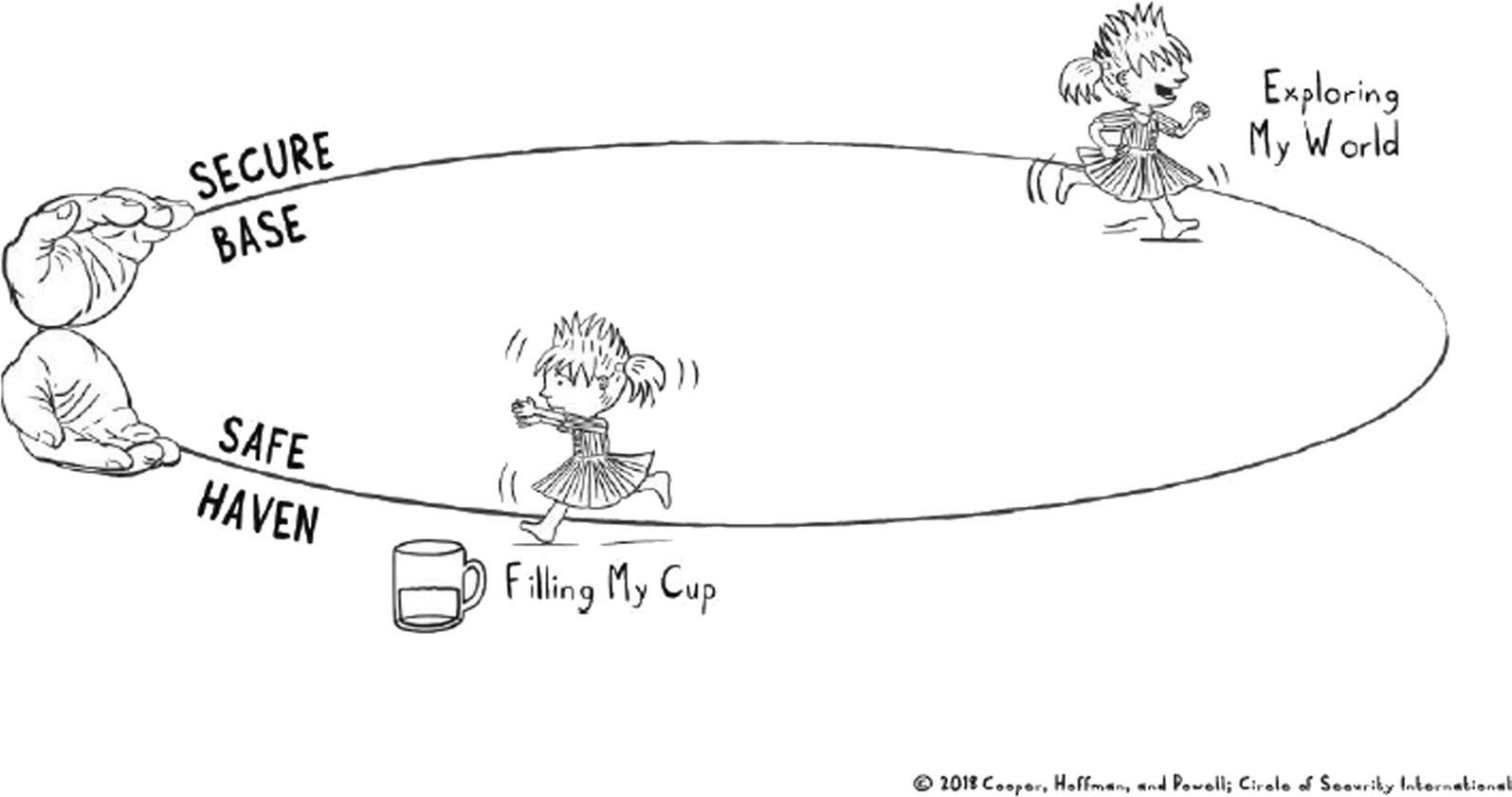
The Circle of Security (from Circle of Security International™)

An important intervention tool in the program is a graphic illustration, the “Circle of Security” (Fig. 1) capturing core asumptions of attachment theory [28]: that children develop a secure attachment relationship if the caregiver provides “a safe haven” (i.e. is available when the child is distressed and needs comfort) and “a secure base” (i.e. is available when the child is exploring and supports the development of new competencies). Througout the intervention, the Circle of Security graphic is used as an observational and analytical framework for the caregivers to explore and reflect on how to support positive development in the children in their care.

**Fig. 2:**
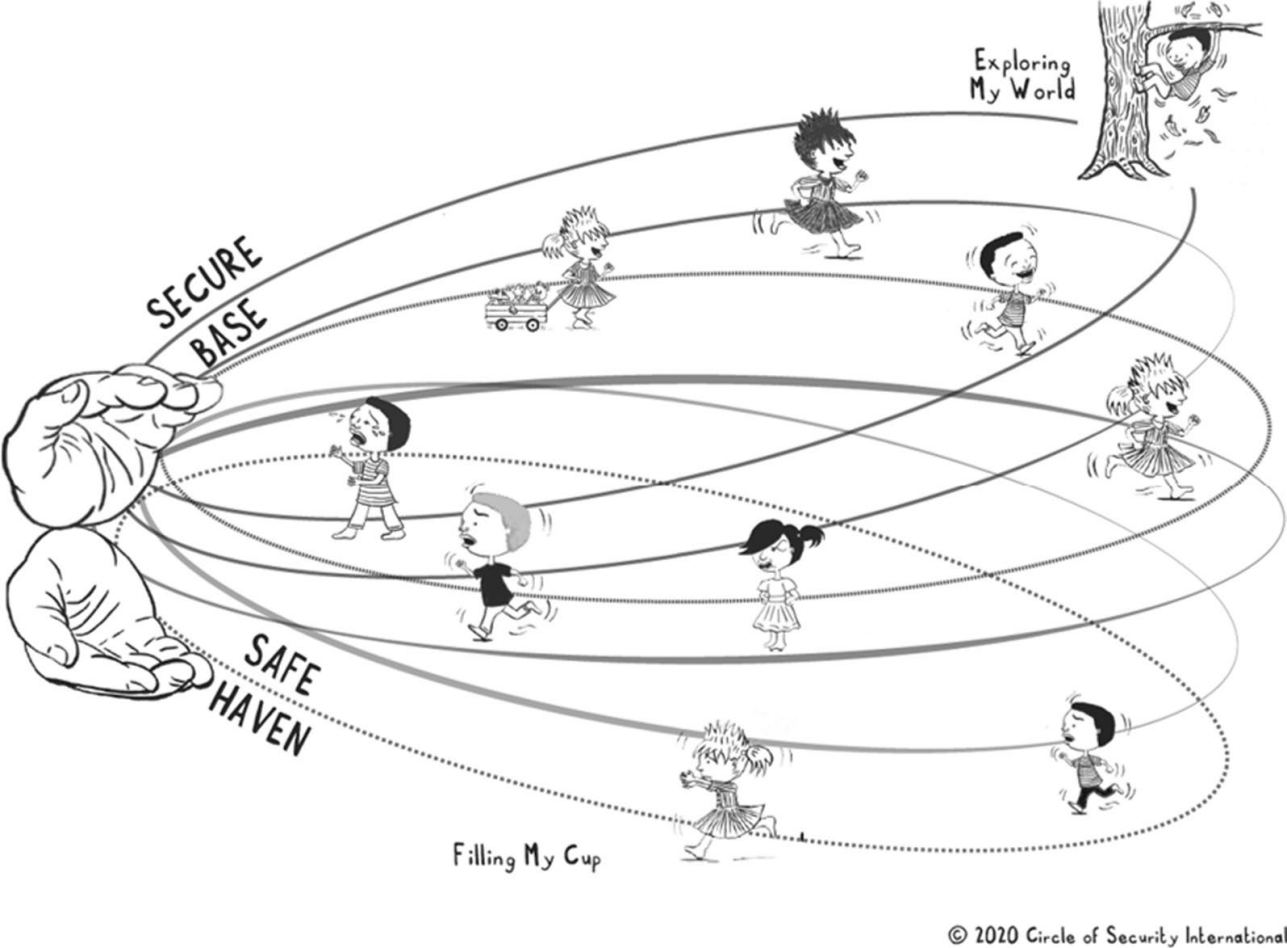
Circle of Security in group-based childcare (from Circle of Security International™)

Figure 2 provides an example of how the material of the intervention has been adapted to capture the reality in group-based childcare, i.e., that the caregiver most of the time must provide a secure base and safe haven for several children who often have differing needs “on the circle”.

The “Circle of Security” also reflects another core assumption from attachment theory: that a child’s exploration, play and learning depends on the the child’s experience of the caregiver as available and responsive to the child’s need for emotional support [28] (“filling up the emotional cup”, see Fig. 1). Therefore, the model assumes that supporting caregivers’ ability to be a secure haven and a secure base is simultaniously supporting child learning and autonomy.

Another core theme of the program is how to be “Secure hands” for the child, i.e. being able to be a caregiver who is “bigger, stronger and wiser” while still being “kind”, referring to the ability to take charge and set limits for children in a sensitive manner, a theme which is of particular importance in the childcare setting where structure and limit setting is a key aspect of process-quality [30, 52].

**Fig. 3:**
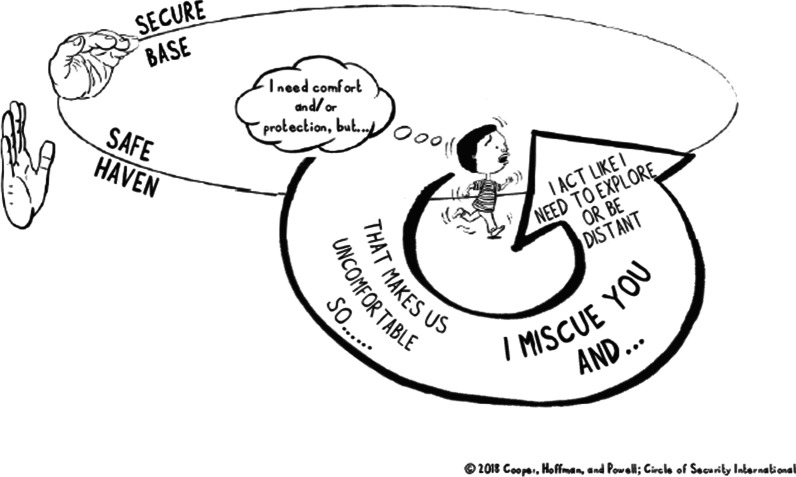
Ilustration of the development of an avoidant strategy in the child (from Circle of Security International™)

To support caregivers’ ability to interpret children’s behavior in terms of emotional needs (mentalizing), and in particular the needs behind the behavioural strategies of insecurely attached children, the notion of ‘cues’ and ‘miscues’ is introduced. Secure children generally communicate their needs clearly (via ‘cues’), for example by seeking closeness or by crying when needing emotion regulation and comfort, or by asking for help when in need of exploration support [26]. Applying the language introduced in the COS approach, insecurely attached children may “hide their needs” and “miscue” caregivers, for example by avoiding the caregiver when they in fact need closeness and comfort (avoidant strategy), or by seeking closeness and being “clingy” when in fact in need of exploration support (resistant/ambivalent strategy)[26]. Figure 3 provides an example of how an avoidant strategy is illustrated in the COS-C program. This theme is of particular importance in terms of facilitating the caregivers’ ability to remain sensitive sensitive towards children who have developed insecure interactive strategies.

An essential part of the program is the use of pre-produced video vignettes of both problematic caregiver-child interactions and interactions where the caregiver is responsive and sensitive. These are used to illustrate how caregivers’ may or may not struggle in meeting childrens’ emotional needs and to faciliate group reflection and learning.

In addition to the adapted visualization of the Circle of Security (Fig. 2), the COS-C program includes material and exercises on “indentifying the invisible children” (e.g., socially withdrawn children), how to support children and parents during check-in and pick-up situations, how to meet the needs of children who are “hard to connect with”, and naming the children’s feelings [51]. Another important adaption focuses on how to facilitate interactive repair between children which is pivotal for fostering positive peer-interactions.

During the sessions, the COS-C facilitator supports the caregivers in using the Circle of Security graphic to reflect on the video material and on examples from their daily interactions with the children in their care. Moreover, the caregivers are invited to reflect on what may prevent them from meeting a child’s needs in specific situations. This joint reflection is hypothesized to be the mechanism of change for the intervention.

While an important goal of the intervention is to facilitate the caregivers’ reflections on children’s mental states, another is to support the caregivers in reflecting on their own mental states and how these might impact their ability to support the child’s changing needs “on the circle”. In doing so, the COS model fits in the category of mentalization-based interventions, wherein understanding of the child’s behavior and the caregiver’s response is organized through recognizing, appreciating and hypothesizing about mental states of the child, but also those of the caregivers themselves [40].

While evidence from parent–child studies suggests that the COS approach can positivly improve caregiver sensitivity, caregiver-child attachment security, and caregiver self-efficacy, [25, 53] research on the COS approach in in the childcare setting is still very limited, though promising. Gray [54] conducted a quasi-experimental pilot study investigating the impact of the COSP intervention with licenced childcare providers (*N* = 34). Findings indicated an increase in the childcare providers’ self-efficicacy corncerning the management of challenging child behaviors compared to a control group not receiving the intervention (*N* = 17). In another study [55], attending a COS training workshop resulted in increased empathy as well as greater understanding of attachment theory-principles among 202 practitioners working with families of young children. However, differences among sample characteristics and intervention format does not allow for comparison of study results and also limits generalizability to other childcare practitioners and work environments. No previous study has investigated effects on observed interaction quality and caregiver mentalization in the professional childcare setting.

#### The moderating role of personal and structural factors

Previous intervention studies of COS-based programs with parents have found that intervention effects may vary for different subgroups [56–59] albeit comparisons and firm conclusons across studies are limited due to the use of dissimilar intervention formats and measures. Yet, to gain insight into the issue of “what works for whom?” in this study, we explore two potential moderators, i.e.professional caregiver attachment and staff stability, of the effect of COS-C—to examine if the intervention affects different subgroups of caregivers differently.

Two previous COS studies with mothers and infants have suggested that caregiver attachment style may moderate effects of COS-based interventions on caregiver sensitivity. One study [58] found that maternal unresolved trauma history moderated the effect of a 20-week COS intervention; mothers with unresolved attachment showed an increase in caregiver sensitivity compared to mothers without unresovled attachment, whereas the opposite was true in the control group. Another study [57] found that maternal attachment avoidance moderated the effect of COSP; after the intervention, mothers with high levels of attachment avoidance in the intervention group had more secure and less insecure-disorganzied children compared to the control group. We will add to this line of research by exploring professional caregiver attachment style as a moderator of the effect of COS-C on caregiver interactive skills.

In a childcare context, it is improtant to not only consider personal but also structural factors, such as staff stability. It is possible that structural factors affect the caregivers’ ability to implement learnings from the COS-C in their daily interactions with the children. While there is mixed evidence that structural factors are related to the quality of caregiver-child interaction [31], based on experiences from piloting of the COS-C, (see below), we consider it relevant to explore whether staff stability (sick leave and staff turnover) moderates the effect of COS-C. The results may inform future childcare centers’ decisions in terms of when to implement interventions targeting caregiver-child interactions.

#### Piloting of the COS-C

We piloted the COS-C with a group of seven professional caregivers and their manager from the same municipality from which participants for the current study are recruited. Information on acceptability and feasibility of the intervention was collected to inform the final protocol. One important learning from the pilot was that the intervention contributed to an increased sense of collegial support and perceived resources to cope with stress [60] and the participants reported that they felt less overwhelmed by managing challenging children and less inclined to call in sick. This is in line with experiences reported by the developers of the model [51] as well as results from a study where caregivers in center-based childcare reported increased resources to cope with work-related stress after participating in an intervention that aimed to improve their interactive skills [61]. By the use of both questionnaires and interviews, we therefore investigate the potential of the COS-C to positively influence caregiver’ perceived job resources and explore their experiences of completing the program with their colleagues.

### Current study

#### Study aims

First, the main aim is to investigate the efficacy of COS-C in enhancing caregivers’ interactive skills when interacting with a group of children in a natural busy real-life setting and their mind-mindedness (MM). That is, we aim to evaluate if the COS-C can positively impact the core aspects of process quality in childcare. Moreover, we investigate the potential of COS-C to positively influence caregivers’ work-specific resources to cope with stress.

Second, building on literature suggesting that the ability to mentalize (here operationalized as MM) is important for caregivers’ sensitive responsiveness; we aim to investigate if changes in the caregivers’ mind-mindedness relates to changes in their interactive skills.

Third, we aim to explore if effects of the COS-C are moderated by personal and structural factors, here operationalized as the caregivers’ own attachment style and staff stability.

Fourth, we evaluate the implementation of the COS-C by examining childcare providers’ experience of the intervention’s acceptability and feasibility, and we will examine the degree to which acceptability and feasibility relates to the intervention’s effectiveness. Investigating feasibility and acceptability of the COS-C intervention is essential in terms of evaluating the potential for up-scaling and further implementation of the intervention in Denmark [62].

Fifth, we employ a qualitative approach to investigate a) how the caregivers and their managers make sense of changes caused by the intervention and b) how the caregivers experience the intervention in terms of its feasibility and acceptability when implemented in their daily practice.

#### Primary hypothesis

- The caregivers in the intervention condition show higher levels of sensitive responsiveness post intervention, compared to the childcare providers in the waitlist control group.

#### Secondary hypotheses

- The childcare providers in the intervention condition
  - show higher levels of mind-mindedness,
  - display higher levels of interactive skills (i.e., respect for child’s autonomy, structuring and limit setting, verbal communication, developmental stimulation, and fostering positive peer interactions), and
  - report more work-specific resources to cope with stress.
- Intervention effects on interactive skills are mediated by changes in the caregivers’ mind-mindedness.
- Intervention effects are moderated by staff stability and the caregiver’s own attachment style:
  - COS-C effects caregivers working in childcare centers characterized by higher levels of staff stability differently than childcare providers working in childcare centers characterized by lower levels of staff stability.
  - COS-C effects caregivers with a secure attachment style differently than caregivers with an insecure attachment style.
- Intervention effects on interactive skills and mind-mindedness are predicted by childcare provider’s experience of the intervention’s acceptability and feasibility.

## Methods

This protocol paper adheres to the SPIRIT guidelines.

### Trial design

The SECURE (SEnsitive Care: Understanding and REsponding) project is a prospective, parallel, 2-year cluster-randomized controlled trial, where teams of caregivers are randomized to either the COS-C intervention or a waiting-list control group.

### Study setting

SECURE is a collaborative project between the Center for Early Intervention and Family Studies at the University of Copenhagen and the municipality of Høje-Taastrup, in the Capital Region of Denmark. This municipality is a well-suited collaborator as the proportion of at-risk and low-SES families is relatively high compared to other Danish municipalities [63].

### Participants and procedure

Participants are caregivers from early childcare centers for children aged 0–2.9 years from Høje-Taastrup.

In February 2020, all managers of the early childcare centers (*N* = 35) in Høje-Taastrup were invited to participate in the project at a meeting held by the head of public childcare in the municipality and the Principal Investigator (PI, JSN). One of these childcare centers volunteered to participate in the pilot project. The remaining 34 center managers signed up for a visit in their center by the PI where their staff, i.e. the caregivers, were invited to participate. Due to the Covid-19 pandemic, some of these meetings were held online. At these meetings, the caregivers were informed about the project, it was made clear that participation was voluntary, and caregivers in each center were asked to consider whether they were interested in participating in the project. Subsequently, the managers gave their notice of participation to the project team.

**Fig. 4:**
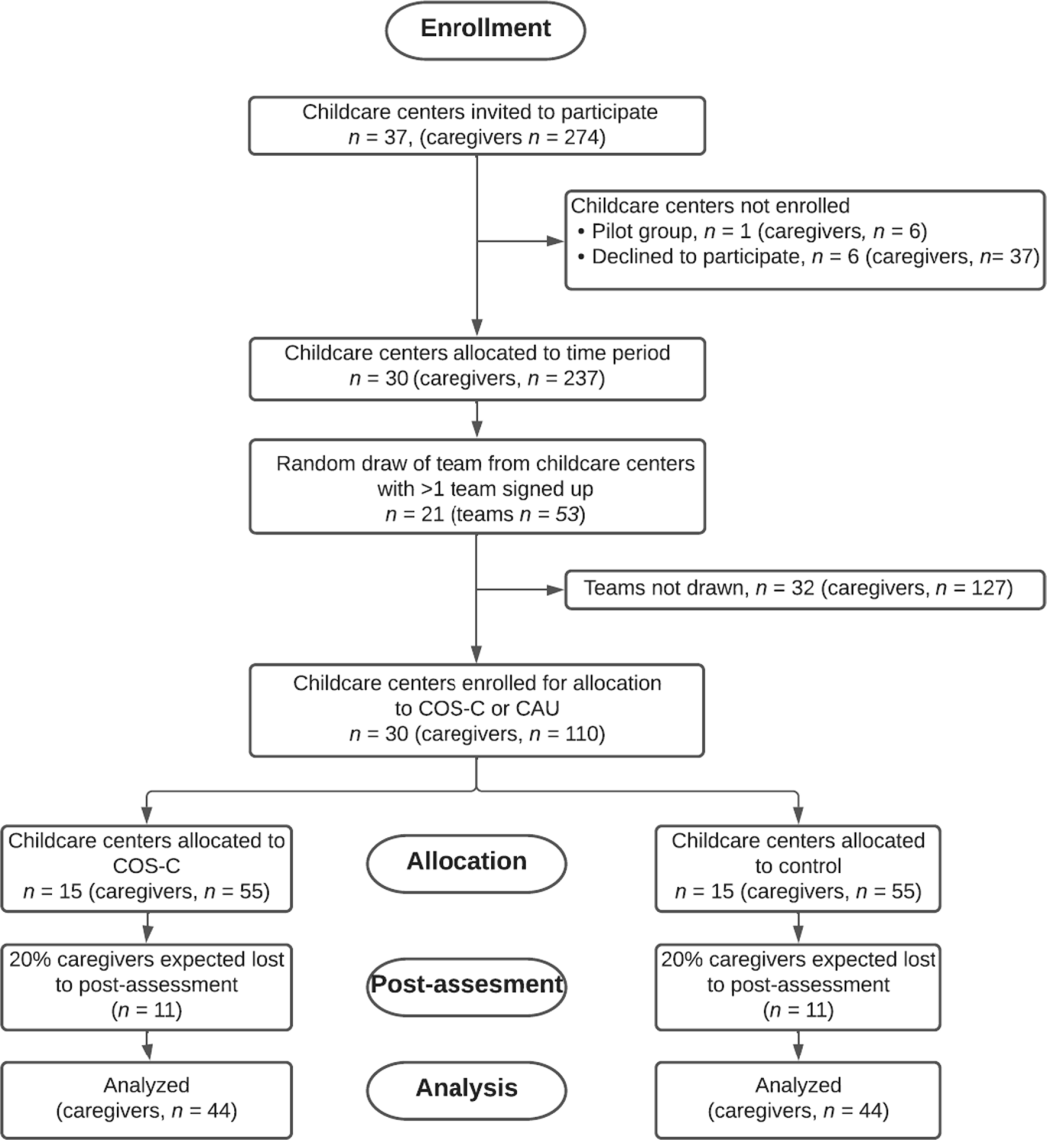
Flow of participants

While 28 of the initially invited childcare centers signed up for participation, six centers declined to participate. In 2021, two new childcare centers were established in the municipality; they were invited using the same procedure as described above and gave their consent for participation primo 2022. Currently, 30 childcare centers have consented to participate with a team, corresponding to approximately 110 caregivers, and all have been allocated to a time-period (see below for randomization procedures). Should more childcare centers (e.g., centers that are established after project start) be interested in participation, they will be informed about the project and enrolled accordingly. Figure 4 provides an overview of enrollment and allocation in the project. Data collection began in April 2021. Currently, participant enrollment and data collection is ongoing.

Caregivers participate in the COS-C program together with their team. A team of caregivers typically consists of three or four caregivers who have 12–14 children in their care, but some teams are larger. Irrespective of the size of the childcare center, each center participates in the study with one team of caregivers. This is mainly due to budget constrains but it also prevents spill-over effects and contamination within the centers. If more than one team from a childcare center is interested in participation, a random draw will determine which team participates (see randomization procedures below). To be eligible for participation, the participants in the team must be 18 or older, employed on a fixed-term contract, and not be part of the management of the childcare center.

A COS-C group consists of 6–10 participants. To make sure that COS-C groups are neither too small nor too large, teams consisting of four or less participants partake in a COSP-C group with a team from another childcare center whereas teams consisting of five or more caregivers constitute their own COSP-C group.

### Randomization

Randomization to the COS-C intervention or waiting-list control condition is done at the center level in a ratio of 1:1 to either COS-C or waiting-list. Randomized allocation is performed by an independent researcher (MP), who has no practical involvement in the trial, using anonymized lists of participant IDs, childcare center IDs and team IDs. Randomization lists are stored in a secured, logged drive. Allocation is performed in four steps:

1. *Random allocation to time period*: Given that not all centers could be trained in COC-C at the same time, the project management decided that each participating childcare center should be randomly allocated to one of four time periods (i.e. spring 2021, autumn 2021, spring 2022, autumn 2022) indicating when caregivers in the childcare center are expected to participate in data collection and the COS-C intervention. This randomization was conducted four months prior to the first project period (primo September 2020) to provide the childcare centers time to plan. Each childcare center was numbered and allocation was done by using a random number generator (random.org) generating a random number between 1 and 4 indicating each of the four time periods. Due to project resources, eight childcare centers are allocated to the first three time periods, and nine childcare centers are allocated to the last time period. When a time period reached the maximum number of childcare centers, no further childcare centers could be added to the time period. The childcare centers with teams consisting of more than five caregivers counted for two childcare centers in the allocation procedure.
2. *Random draw of one team from each childcare center*. Minimum three months before the beginning of a time period, the centers allocated to the time period are required to confirm the number of teams who have consented to participate. If more than one team has consented, a random draw determines which of the teams are going to participate in the project. The teams are numbered, and allocation is conducted by using a random number generator (random.org) generating a random number (e.g. from one to three if three teams have consented) indicating which team is the participating team. The childcare center is then informed about which of the teams will participate in the trial.
3. *Random allocation to COS-C or waiting-list group.* The last randomization is to decide which childcare centers receive COS-C in the study period and which are allocated to the waitlist (control groups). Each childcare center has a unique ID, and for each childcare center, a random number between 0–1 is generated to determine whether the team is allocated to COS-C or the waiting-list group.

### Intervention

The intervention group (50% of the sample, randomly selected) will receive the COS-C intervention between the baseline (T0) and first post-assessment (T2). In this project, the intervention consists of eight weekly two-hour group sessions with 6-8 professional caregivers per group. Througout the intervention, the caregivers are presented to key principles from attachment theory. The first two sessions introduce the model’s framework, i.e. the Circle of Security (Figs. 1, 2) and focus on observational skills and ability to infer children’s attachment and exploratory needs from their behavior. Each following session will then introduce new themes for reflection and learning, such as child emotion regulation and co-regulation and how the caregiver’s own relationship history may influence their caregiving practices, understanding difficult child behavior (“miscues”, Fig. 3) and interactive repair.

The sessions are facilitated by psychologists from the research team who are all trained and certified in the COS-P and COS-C intervention [25, 64]. The psychologists receive ongoing supervision provided by a certified COS-P fidelity coach (the first author).

To support the implementation of COS-C principles, managers of each participating team are invited to participate in the sessions together with their staff. If the managers choose to particpate, they are instructed to participate on equal terms as their staff. Group sessions take place either in the caregivers’ own childcare center or in another avaible location, such as other suited rooms in the municipality. The Municipality ensures that the childcare centers can call in substitutes for the caregivers while they attend COS-C. The COS-C material (i.e. manual handouts and exercises) that is added to the COSP program has been translated into Danish, and is used together with the Danish version of the COSP video-material [65].

### Control condition

Caregivers in the waiting-list control group take part in the T0 and T2 assessments parallel to the intervention group but they do not receive any intervention between asessments, and they are not in any contact with the project team other than for planning and handling practical issues. After T2 data collection, participants in the waiting-list control group attend a COS-C group (T3), followed by a post-intervention assessment with the waiting-list control group only (T4).

### Measures

An overview of the primary and secondary outcome measures is provided in Table 1.

**Table 1:** Overview of measures used in the study

	Measure	Time point	Name of instrument	Type
Primary outcome	Caregiver sensitive responsiveness	T0, T2	Caregiver Interactive Profile Scales (CIP-scales [52])	Observation based on video-recordings
Secondary outcomes	Mind-mindedness (representational)	T0, T2	Mind-Mindedness Interview (MM-interview [68])	Interview, coding based on transcript
	Mind-mindedness (interactional)	T0,T2	Mind-Mindedness [67]	Observation, coding based on video-recordings and transcripts of the video
	Caregiver Respect for autonomy, Structure and limit setting, Verbal com-munication, Developmental stimulation, & Fostering positive peer interactions	T0, T2	Caregiver Interactive Profile Scales (CIP-scales [52])	Observation based on video-recordings
	Caregiver resources to cope with work-related stress	T0,T2	The Child Care Worker Job Stress Inventory [69]	Questionnaire
Other measures	Caregiver attachment style	T0	Experiences in Close Relationships – Revised Version (ECR-R [70])	Questionnaire
	Structural factors	T0, T2	Questionnaire designed for the purpose of the study by The Danish Evaluation Institute	Observation and questionnaire
	Acceptability	T0, T2, T4	The Acceptability of Intervention Measure (AIM, [71])	Questionnaire
	Feasibility	T0, T2, T4	The Feasibility of Intervention Measure (FIM, [71])	Questionnaire
	Experiences of the COS-C	T2, T4	The Client Change Interview (CCI) [72]	Interview, semi-structured
	Demographic factors	T0	Developed for the project	Questionnaire

#### Primary outcome measure

*Caregiver Interactive Profiles Scales (CIP-scales*) [52] are used to assess the caregivers’ interactive skills at T0 and T2. This is an observational instrument developed to assess the quality of caregivers’ interactive behavior in center-based childcare with 0–4 year-old children, and has been used to assess the quality of caregiver-child interactions in Western [30, 52] and non-Western childcare settings [66]. Ratings are based on filmed observations of the caregivers in their natural childcare setting where they typically interact with groups of children. To capture a broad range of situations with differing demands, the caregivers in this project are observed in four different situations: diaper change, lunch/snack time, free play, and transition between activities (10 min each). The caregivers are not given any specific instructions and are asked to follow the regular program to capture a usual day.

CIP-scales consists of six scales: (1) Sensitive responsiveness, (2) Respect for autonomy, (3) Structure and limit setting, (4) Verbal communication, (5) Developmental stimulation, and (6) Fostering positive peer interactions. Each interactive skill is rated on a 7-point scale with higher scores indicating higher quality of the skill. Sensitive responsiveness is used as the primary outcome and is the core experimental variable in this study.

The coding manual provides definitions of each interactive skill and describes what characterizes each score. For example, a score in the low (1, 2) range of Sensitive responsiveness is characterized as: “*Hardly provides emotional support to the children. She misses many signals or her reactions are too slow or inadequate. She may show indifferent or detached behavior*” and a score in the high (6, 7) range is described as: “*Shows warm and genuine interest in the children and provides emotional support when needed. In general, the caregiver responds promptly and appropriately to the children’s signals, thereby functioning as a *“*secure base*”* for the children. If unable to respond, she acknowledges having noticed the signal and provides a more complete response as soon as possible*” ([52] p. 778).

The videos will be rated by a trained observer blind to group allocation and with no practical involvement in the intervention and no prior knowledge of the participants. To calculate interrater reliability, 20% of the observations are selected randomly and coded by a second coder blind to group allocation and the primary coder’s ratings. Coders will be trained by the developer of the CIP-scales, Katrien Helmerhorst (sixth author), and will attend regular meetings to check for adherence to the coding principles and prevent drifting. The coding manual has been translated into Danish by The Danish Evaluation Institute (EVA) in collaboration with the developers of the scale at Netherlands Consortium Kinderopvang Onderzoek.

#### Secondary outcome measures

*Mind-Mindedness* is the second core outcome of the study. MM can be assessed in two ways. “Offline” mentalizing, or representational MM, is assessed by using a well-established, simple interview, addressing the caregivers’ tendency to spontaneously think of the child as a psychological being [67]. “Online” mentalizing, or interactional MM, is assessed by observing the caregiver’s ability to apply a mentalizing stance towards the children during interaction. The interview approach is proposed to assess the representational aspect of MM, i.e. how the caregiver keeps the child in mind in absence, whereas interactional MM is proposed to be a construct at the interface of representational mentalizing and caregiver sensitivity, as it is an interactional construct of “enacted” mentalizing [42]. To capture both interactional and representational MM, two different measures are applied at T0 and T2 for both groups.

*The Mind-Mindedness interview* [68] is used to asses representational MM. We adapted the MM interview to a childcare setting by asking the caregiver to think about two children, whom they perceive as challenging, and are then given an open-ended question: “*Can you describe [child] for me?*”*.* The caregiver is initially informed that there are no right and wrong answers, and that they should talk about the first thing that comes to mind. If they ask for guidelines for answering the question, it is repeated that no specific type of description is required and that they should talk about what comes to mind. After this, a follow-up question is employed: “*Can you say anything else about [child]?*”. The recorded interview is transcribed verbatim, and according to the manual [68] each attribute that refers to the child is classified into one of the following categories: (1) Mental attributes, (2) Behavioral attributes, (3) Physical attributes, (4) General attributes, (5) Self-referential comments and (6) Comments regarding institutional practices. To control for differences in verbosity, representational MM is calculated as the proportion of mental attributes, i.e. number of mental attributes divided by total number of attributes produced by the caregiver.

*Interactional MM* is assessed by observing the caregiver’s use of appropriate mind-related comments during interaction with the children in the childcare center, and thus represents the ability to envision mental states in the children from the children’s behavior and to convey such understanding verbally to the children. Twenty minutes of video-recorded interactions between the caregivers and the children in their care will be used for coding of MM, following the procedure from a previous study [47]. Each video is transcribed verbatim, and then coded following the MM coding manual [67]. First, mind-related comments are identified i.e. if they refer to the child’s thoughts, intentions or feelings. Next, these comments are coded into appropriate or non-attuned comments upon watching the video. Comments are coded as appropriate mind-related comments if (a) the coder agrees with the caregiver’s interpretation of the child’s mental state; (b) the comment links the current activity with similar events in the past or future; (c) the comment refers to the child’s preferences; (d) the comment clarifies how to proceed after a lull in the interaction.

Comments are coded as non-attuned if (a) the coder disagrees with the caregiver’s interpretation of the child’s mental state/s; (b) the caregiver refers to a physical state without overt signs of such; (c) the caregiver refers to a past or future event unrelated to the child’s current activity; (d) the caregiver asks what the children/child want/s, or suggests a new activity when the child is already engaged with something else; (e) the caregiver seems to be attributing internal states that were not implied by the child’s behavior and thus appeared to be projections of the caregiver’s own internal state; and (f) the referent of the comment is not clear. We will code for caregiver mind-mindedness in a group of children, i.e. differentiating between dyadic and non-dyadic mind-mindedness, following Colonnesi and colleagues’ adaptation to the original mind-mindedness coding scheme [48].

*CIP scales* [52] (described above) other than Sensitive responsiveness. i.e., Respect for autonomy, Structure and limit setting, Verbal communication, Developmental stimulation, and Fostering positive peer interactions are used for assessing caregiver interactive skills, as secondary outcomes. All interactive skills are measured with the CIP scales in both groups at T0 and T2. *The Child Care Worker Job Stress Inventory* [69] is used to test the effect of the COS-C on the caregivers’ work-specific resources to cope with stress (e.g. feeling they are helping children develop, feeling close with children, feeling their work is valued). The instrument has demonstrated high validity and internal consistency and is developed specifically for childcare workers in both center- and home-based settings. It consists of three scales out of which we use the Job Resources Scale. This scale consists of 17 questions that are rated on a 5-point Likert scale from Rarely/never (= 1) to Most of the time (= 5). Higher scores reflect more access to resources to manage job-related stress. The questionnaire has been translated and adapted to the Danish context by the first and last author for the purpose of this study, and face validity of the questions was tested on a pilot group of eight caregivers. The measure is part of a online survey that is distributed to participants in both groups at T0 and T2.

#### Moderators, baseline measures, and covariates

*Experiences in Close relationships-Revised Version, ECR-R* [70] is used to assess the caregivers’ own attachment style in terms of intimate, adult relationships. The ECR-R is the most frequently self-report measure of adult romantic attachment dimensions [73] and has been found highly reliable [74]. The scale consists of 36 items and measure attachment on two dimensions: (a) attachment avoidance (fear of intimacy and interpersonal dependence) and (b) attachment anxiety (fear of abandonment and a craving for interpersonal closeness). Avoidance and anxiety are continuous dimensions with attachment security defined as the absence of both. In some studies, the attachment avoidance and anxiety dimensions are highly correlated [73], and in this case, the ECR-R can be used as a one dimensional measure of attachment security by collapsing the two dimensions; security is then represented as low avoidance and anxiety, whereas insecurity is represented as high avoidance and anxiety [75]. ECR-R is part of an online survey that is distributed to both groups at T0.

*Structural factors*: Child–adult ratio is observed and noted by the observers who video-record the caregiver-child interactions in both groups at T0 and T2. Staff stability, child–adult ratio and grouping is assessed by online survey at T0 and T2 in both groups. Other factors regarding staff stability (e.g. sick leave, new hires, and use of substitutes) are measured via online surveys filled in by the managers of each childcare center at T0. Caregiver age, educational levels, and other background variables are measured by online survey filled in by the caregivers in both groups and at T0. The questionnaires used to measure structural factors were developed by The Danish Evaluation Institute (EVA) for other large scale evaluations of the quality of Danish childcare [76] and adapted by EVA for the purpose of this study.

#### Evaluation of feasibility and acceptability

To evaluate the feasibility of the intervention and how the participants experience the intervention in terms of its contribution to childcare practice, the following measures will be applied:

*The acceptability of intervention measure (AIM) *[71] is used to assess the caregivers’ and managers’ acceptability of the COS-C intervention. The AIM consists of four questions asking whether participants likes and welcomes the intervention and whether it meets their approval and is appealing to them.

*The feasibility of intervention measure (FIM) *[71] is used to measure whether the caregivers and their managers consider the COS-C intervention to be feasible to implement in a Danish early childcare context. The FIM consists of four questions asking whether the intervention seems implementable, possible, doable, and easy to use in practice.

The FIM and AIM are translated by the first and last author and piloted on a group of caregivers. AIM and FIM are distributed in both groups at T0 using an online survey, at T2 for the intervention group only, and at T4 for the waiting-list control group only.

*Client change interview (CCI)* [72, 77] will be used to assess how the participants experience the intervention, and is conducted individually with each caregiver and manager, who have participated in COS-C. The CCI will be conducted at T2 with the intervention group and at T4 with the waiting-list control group. The CCI is a semi-structured qualitative interview originally developed to help clients express their experience of therapy as freely as possible. The interview guide has been adapted to the childcare context for the purpose of this study. The caregivers are initially asked an open question where the they can speak freely about their experience of the intervention. Afterwards questions address changes experienced by the caregivers following the intervention, possible causes of these changes, aspects that were helpful and hindering and the experience of the research project. The interviews are audio recorded and verbatim transcribed using the software NVIVO.

### Sample size and power

The primary outcome for the study is the childcare providers’ sensitive responsiveness using the CIP-Scales [52]. Therefore, sample size was determined as the number of participants required to achieve min. 80% power for detecting a change in this measure with a two-sided alpha of 0.05. Two previous RCTs have examined interventions aiming at increasing professional caregivers’ sensitive responsiveness using the CIP-Scales. These studies find medium (*d* = 0.55) to large (*d* = 0.72) effects of the interventions on caregivers’ sensitive responsiveness post intervention. For normally distributed outcomes, a sample of 29 day care centers (15 intervention, 14 control corresponding to 110 caregivers) with each 3.4 caregivers yields a statistical power of 81% to detect an effect size of *d* = 0.55 using a two-sided alpha of 0.05, a correlation between baseline and post-intervention of 0.5 and an ICC of 0.1. This is the conservative estimate using the lowest effect size. If the effect size is 0.72 the statistical power rises to 96%.

## Discussion

It is well-documented that the quality of the interactions that children have with their professional caregives impacts their development across various important domains well beyond the first years of life [5, 10, 11]. Particularly children from at-risk backgrounds are found to benefit from high quality childcare in various developmental domains [9, 18–20]. Therefore, caregivers’ ability to understand and respond to the children’s needs, also when children do not communicate their needs clearly is pivotal. This study protocol presents the first RCT testing the efficacy and feasibility of the COS-C, an attachment- and mentalization-based intervention originally developed as a parenting program but adapted to the childcare context. The setting is Danish center-based childcare with children aged 0–2 year.

Overall, it is hypothesized that the intervention will enhance caregivers’ sensitive responsiveness and mind-mindedness as well as other dimensions of caregiver interactive skills. Although a previous quasi-experimental study has shown promising results in terms of supporting childcare providers’ self-efficacy and empathy using the Circle of Security approach [54], it has not been examined whether these effects translate into professional caregivers’ sensitive behavior and mind-mindedness towards the children in their care.

### Strengths and limitations

The most important strength of the study is the randomized controlled design. While previous quasi-experimental studies have shown promising results in terms of using the Circle of Security-approach for professional development [54], no previous studies have examined this in a randomized controlled trial. Furthermore, in a meta-analysis on the effects of interventions in childcare, the authors concluded that although interventions targeting caregiver-child interactions are moderately effective, we need more studies with sufficient power and high-quality measures [24]. This study contributes to the literature in both of these respects:

First, we conducted an a priori power analysis to determine sample size in the present study, and compared to the studies in the aforementioned meta-analysis, this study is among the largest studies examining the effects of interventions targeting childcare providers’ interactive skills. Second, we use well-validated, reliable and theoretically sound measures for capturing changes in childcare providers’ interactive skills [52] and mind-mindedness [42]. In particular, the use of an observational measure for capturing childcare providers’ interactive skills during naturalistic situations is an important merit of the present study. Furthermore, the use of observational measures rated by blinded coders reduces the bias associated with self-report.

Third, apart from evaluating the effectiveness of the COS-C, the present study also aims at examining the mechanisms of change. In a mediation analysis, we will examine whether the COS-C improves caregiver’s interactive skills through supporting their mentalizing abilities, here operationalized as caregiver mind-mindedness. This knowledge will contribute to the development of interventions targeting childcare providers’ interactive skills, and since few studies have examined the links between mentalizing and interactive skills in professional caregivers [47], the knowledge will also contribute to our theoretical understanding of factors underlying professional caregivers’ sensitive and insensitive responses to children in their care.

Fourth, the mechanisms of change and the implementation of the COS-C are examined using a mixed-methods approach. Acceptability and feasibility is measured using validated measures [71] and the questionnaires are combined with an adapted version of the qualitative semi-structured Client Change Interview (CCI), which has been used extensively to study client’s experience of therapy-processes [72]. The qualitative approach provides in-depth information about mechanisms of change and implementation of the COS-C in a Danish center-based childcare setting, and allows for hypothesis generation. We believe that the combined qualitative and quantitative results will contribute to the development of interventions for childcare providers in general, and further adaptation of the COS-C to the childcare setting. Also, a focus on implementation is a considerable strength because research-based interventions might not be effective in real-life practices when implementation is unsuccessful [62].

Fifth, it is a strength that the study is conducted in a municipality where the proportion of at-risk and low-SES families is relatively high compared to other Danish municipalities. However, this strength also implies some limitations as the specific characteristic of this study sample limits generalization of results to populations with a different SES background. Yet, given the findings of reviewed literature herein, it is important to investigate the effects of the COS-C in a more at-risk population where the children are particularly vulnerable for developing insecure attachments with their professional caregivers with subsequent consequences for long-term developement [33].

Finally, randomization is conducted at the center level, ensuring that teams in the intervention group and waiting-list control group come from different childcare centers, thereby preventing a potential “spillover” effect from the intervention group to the control group.

The study also has some limitations. First, as the caregivers all work within the same municipality, there is a risk of information flow about the intervention principles from the intervention group to the waiting-list control group. However, the randomization process is designed to reduce such biases by ensuring that allocation to the intervention is conducted at the center level.

Second, considerations should also be made in relation to the choice of a waiting-list control group because effect size estimates have been found to vary depending on the control condition, and waiting-list control groups generates larger effect sizes than no treatment or psychological placebo in psychotherapy research [78]. Whether this applies for interventions tested in the childcare context is unknown. The waiting-list design also makes it impossible to collect follow-up data to examine long-term effects. Nevertheless, if this trial shows an effect of COS-C, future studies would benefit from comparing COS-C to other interventions targeting caregiver-child interaction in childcare or modified interventions of the COS-C, e.g. COS-C with added video feedback. These studies would shed light on what components are most effective in supporting childcare providers’ interactive skills.

Finally, and most importantly, we do not measure effects of the intervention on child outcomes. Thus, in the case that the intervention improves childcare providers’ interactive skills and/or mind-mindedness, we will not know the extent to which these effects translate into positive child outcomes. Meta-analytic evidence shows that previous interventions targeting caregiver-child interactions in childcare show moderate effects on outcomes related to childcare providers and only small effects on child outcomes [24]. An evaluation of the effects of COS-C on child well-being and developmental outcomes would require a different design, a larger sample, and importantly, more extensive data collection. Before conducting such a resource-extensive study, we believe that it is important to establish the effect of COS-C on caregiver outcomes. Moreover, as child outcomes and caregiver-child attachment quality is directly linked with caregiver interactive skills [11, 33], we consider testing effects on caregiver MM and sensitivity a first important step. If COS-C shows an effect at the caregiver level, future studies should examine the effect of COS-C on caregiver-child attachment quality and child outcomes.

In conclusion, the presented RCT will evaluate the effect, mechanisms of change, and implementation of COS-C, an attachment-based group intervention aiming at promoting caregiver-child sensitivity and Mind-Mindedness in a Danish early childcare setting. The results of the study will provide insight on if offering a systematic manualized short-term intervention can improve the quality of care offered in Danish childcare centers, potentially informing policy makers on how to ensure high-quality childcare, which is of great importance for children’s current and long-term wellbeing and development.

## Data Availability

Data sharing is not applicable to this article because the study is still ongoing. The study is conducted in a relatively confined sample of childcare providers in an identifiable setting. For reasons of ensuring optimal anonymization of the study participants, datasets will not be made publicly available. Once data collection and coding is finalized, the datasets used and/or analyzed during the study will be available from the corresponding author on reasonable request.

## Abbreviations

CCI: Client Change Interview
CIP: Caregiver Interactive Profile
COSP: Circle of Security Parenting™
COS-C: Circle of Security-Classroom™
MM: Mind-Mindedness
MMI: Mind-Mindedness Interview
SECURE: SEnsitive Care: Understanding and REsponding
